# Mesenchymal Stem Cell-Derived Exosomes and Their Therapeutic Potential for Osteoarthritis

**DOI:** 10.3390/biology10040285

**Published:** 2021-04-01

**Authors:** Gi Beom Kim, Oog-Jin Shon, Min-Soo Seo, Young Choi, Wook Tae Park, Gun Woo Lee

**Affiliations:** 1 Department of Orthopedic Surgery, Yeungnam University College of Medicine, Yeungnam University, Medical Center, 170 Hyonchung-ro, Namgu, Daegu 42415, Korea; donggamgb@hanmail.net (G.B.K.); maestro-jin@hanmail.net (O.-J.S.); gom2ya@gmail.com (W.T.P.); 2 Laboratory Animal Center, Daegu-Gyeongbuk Medical Innovation Foundation (DGMIF), Daegu 41061, Korea; msseo@dgmif.re.kr; 3 Department of Orthopedic Surgery, Kosin University College of Medicine, Kosin University Gospel Hospital, 262 Gamcheon-ro, Seogu, Busan 49267, Korea; yuzo0n@naver.com

**Keywords:** mesenchymal stem cell, extracellular vesicle, mesenchymal stem cell-derived exosome, osteoarthritis

## Abstract

**Simple Summary:**

Although mesenchymal stem cells (MSCs) have demonstrated their therapeutic potential for osteoarthritis (OA) treatment in preclinical and clinical studies, conventional MSC-based therapies have some limitations that must be overcome. Extracellular vesicles (EVs) are bilayer membrane structures containing bioactive components including proteins, lipids, and RNAs. EVs are classified into exosomes, microvesicles, and apoptotic bodies according to sizes, origins, biomarkers, and compositions. It has been reported that MSC-derived exosomes contain a variety of cytokines, growth factors, and microRNAs, and have comparable anti-inflammatory and regenerative potentials similar to those of MSCs. Here, we review the characteristics and isolation techniques of MSC-derived exosomes and their use for the treatment of osteoarthritis (OA).

**Abstract:**

Exosomes are nano-sized vesicles (50–150 nm in diameter) that contain nucleic acids (e.g., microRNA and messenger RNA), functional proteins, and bioactive lipids. They are secreted by various types of cells, including B cells, T cells, reticulocytes, dendritic cells, mast cells, epithelial cells, and mesenchymal stem cells (MSCs). They perform a wide variety of functions, including the repair of damaged tissues, regulation of immune responses, and reduction in inflammation. When considering the limitations of MSCs, including issues in standardization and immunogenicity, MSC-derived exosomes have advantages such as small dimensions, low immunogenicity, and lack of requirement for additional procedures for culture expansion or delivery. MSC-derived exosomes have shown outstanding therapeutic effects through chondro-protective and anti-inflammatory properties. MSC-derived exosomes may enable a new therapeutic paradigm for the treatment of osteoarthritis. However, further research is needed to prove their clinical effectiveness and feasibility.

## 1. Introduction

Osteoarthritis (OA) is a common pathology associated with human aging, and its pathogenesis is characterized by synovial inflammation, cartilage degradation, subchondral bone sclerosis, and osteophyte formation, which result in disability [1,2]. OA management has become popular with increasing life expectancy, and has advanced from a symptomatic approach to a fundamental concept. Despite the diversity of approaches for OA management, an optimal treatment capable of reversing the progression of OA has not been established.

Pathophysiologically, OA is a degenerative condition that is initiated with minor trauma and inflammation in the matrix and chondrocytes of the cartilage. In addition, when endplate fissure or trauma beneath the cartilage layer is present, blood vessels grow inside the cartilage layer, leading to the localization of inflammatory cytokines in the cartilage [3,4]. This aggravates the early phase of cartilage inflammation, and eventually, osteoarthritic change progresses.

To establish disease-modifying strategies, it is necessary to understand the molecular and cellular mechanisms of OA that have been identified to date. In the early inflammatory phase, innate immune cells, such as natural killer (NK) cells or macrophages, can play a significant role [5]. Additionally, inflammatory cytokines such as tumor necrosis factor (TNF)-α and interleukin (IL)-1β have a catabolic function, resulting in cartilage degradation [6,7]. As the levels of pro-inflammatory cytokines such as IL-1β or TNF-α increase, the expression of growth factors such as transforming growth factor (TGF)-β increases, matrix metalloproteinases (MMPs) are activated, and ultimately, chondrocyte senescence can be observed [3,4,8].

Some trials at the cellular or molecular level have been developed as a fundamental approach for OA management, mainly using stem cells from various donor tissues, such as bone marrow, adipose tissue, and embryonal tissue [9,10,11]. In particular, mesenchymal stem cell (MSC)-based therapy has been highlighted as a promising approach for OA treatment and has been widely studied in the last few years. MSCs are multipotent progenitor cells that can be obtained from various tissues (bone marrow, adipose tissue, synovium, peripheral blood, and umbilical cord) [12,13]. They have the potential to differentiate into tissues of mesodermal lineage, such as cartilage, bone, fat, muscle, meniscus, and ligaments, in specific environments [14]. Several MSC-related studies have reported encouraging outcomes of inflammation and pain reduction in patients with OA [11,15,16]. MSCs may reduce the secretion of inflammatory factors such as IL-1β, IL-6, IL-8, MMP-1, and MMP-13 in osteoarthritic joints [17,18]. Moreover, they mediate cartilage repair and scar tissue formation by promoting the secretion of several growth factors, such as epithelial growth factor, insulin-like growth factor-1, fibroblast growth factor, TGF-β, and vascular endothelial growth factor [17]. These therapeutic functions contribute to the paracrine and immunomodulatory effects of anti-inflammatory and chondroprotective mediators secreted by MSCs [19,20]. Although it has long been known that MSCs produce abundant growth factors and cytokines, some studies have reported that injected MSCs are largely cleared or caught, and hence, only partially act in the target tissue [21,22]. Moreover, standardization is insufficient owing to the heterogeneity of strategies of MSC isolation and expansion from different tissues [23]. Despite these limitations, several MSC-related studies have reported the outcomes of short-term effects [9,11,16,24]. However, there have been few studies that demonstrate the optimal therapeutic effect of MSCs at the preclinical level as well as in clinical settings.

Trials with MSCs have shown unsatisfactory outcomes in in vivo/in vitro experiments and clinical studies [25,26,27]. The main issues related to the poor outcomes upon the use of MSCs have been documented, as follows. Firstly, MSC engraftment to the target organ, especially the cartilage and some musculoskeletal systems, is very low [25,28]. Several studies have also demonstrated that even if engraftment is good, the MSCs can disappear within several days [29,30,31]. The longevity issue of MSCs may result from certain circumstances, including a poor environment for MSC survival, extreme immunological conditions for the inflammatory state of OA (especially the active phase of OA), and insufficient intercellular communication for signaling between specific cells to better enable the inherent properties of MSCs [32]. Secondly, host cells or tissues exhibit immunological tolerance toward implanted MSCs, and some studies have shown that this tolerance may also be a potential risk for malignancy [33]. MSCs have the capability to adapt their immunological function in an inflammatory environment with the aim of escaping, e.g., killing by NK cells; however, it remains to be determined whether this function poses a risk to immunological cell control when MSCs are applied to an inflammatory milieu [34]. Thirdly, the activity of MSCs can work conversely, in terms of controlling inflammation. In general, MSCs exhibit anti-inflammatory activity at target lesions as well as in other areas. In some cases, on the contrary, they serve to induce pro-inflammatory activity to attract innate immune system and inhibit anti-inflammatory activity in the initial phase of MSC implantation [35]. Accordingly, MSCs exhibit prominent plasticity, exerting both pro- and anti-inflammatory phenotypes depending on their cellular environment [36,37]. After pooling pro-inflammatory activity with the process, MSCs promote a defense response with anti-inflammatory efficacy; hence, a gap between the two contrary activities must be present. During the process, the levels of MSCs and favorable cytokines in the site diminish, causing their function to be restricted. Through this balancing action, MSCs can be the regulation center of immune control and tissue regeneration [38]. In this process, the interactions between MSCs and macrophages are mediated. Macrophages exhibit two phenotypes (M1, pro-inflammatory; and M2, anti-inflammatory), which are mainly related to the secretion of cytokines and the expression of cell surface markers [38,39]. In some cases, modulation of these macrophage phenotypes also serves as a beneficial strategy to maximize the potential of MSCs for efficient cartilage repair [40].

Intercellular communication is essential for maintaining cellular function and supporting homeostasis in multicellular organisms. The communication between cells is regulated by direct contact between them or by mediating secretions such as soluble mediators and extracellular vesicles (EVs). EVs play a significant role in intercellular communications as carriers with critical signals, such as various RNAs, cytokines, and specific proteins. All types of EVs have some functions in intercellular communication; however, among them, exosomes have been highlighted more than other EVs, such as microvesicles and apoptotic bodies, in consideration of their stability, contents, and higher performance [41,42]. Exosomes can originate from a variety of body fluids, such as blood, saliva, plasma, urine, and amniotic fluid, and a variety of cells, such as fibroblasts, epithelial cells, blood cells, adipocytes, neurons, stromal cells, tumor cells, chondrocytes, and MSCs [43,44]. The biology, functions, and role of MSCs depend on their cellular origin and status of the donor cells, especially in relation to the time of exosome generation [42]. Recently published studies have shown that exosomes play a significant role in various processes, such as angiogenesis [45,46], antigen presentation [47], regulation of apoptosis [48], maintenance of homeostasis [49], regulation of inflammation [50], and intercellular signaling [42]. In addition, the potential of exosomes is comparable to or greater than that of MSCs, and their action is facilitated by intercellular communication that integrates complex signals in multicellular organs [51]. Exosome-mediated signaling induces inflammatory responses by delivering various molecules, such as RNAs, proteins, and lipids [42]. Simultaneously, exosomes play a vital role in regulating inflammation in immune responses and reactions [52], because uncontrolled excessive inflammation can lead to tissue or cell damage, causing several pathological conditions. Lastly, several studies have proven that exosomes can induce the entire process of development and differentiation of MSCs [53]. A recent study by Narayanan et al. [54] revealed that pro-osteogenic exosomes isolated from cell culture induce lineage-specific differentiation of innate MSCs in vitro and in vivo. Moreover, MSC-derived exosomes showed a positive effect on RNA signaling and alteration, promoting osteogenic and chondrogenic differentiation [55].

According to the literature, exosomes play a significant role in several biological processes, including intercellular communication, immune reaction and regulation, and initiation and promotion of the development and differentiation of MSCs. Exosomes are also a valuable option for OA treatment. Herein, we summarize the pathophysiology and therapeutic targets of OA, and discuss EVs, especially focusing on exosomes and their therapeutic potential in OA management.

## 2. Why Should We Pay Attention to MSC-Derived Exosomes for OA Management?

Advances in nano-sized fractionation studies that identify active components in media within the 50–200 nm range have been used to characterize biologically active substances such as exosomes [56]. Some studies have demonstrated that MSC-mediated growth factors and cytokines are transmitted by MSC-derived EVs [57].

MSC-derived EVs are heterogeneous populations of particles with different characteristics, and are involved in intercellular communication [58,59]. Depending on the size, expression of membrane markers, and biogenesis, they consist of three main components: exosomes, microparticles or microvesicles, and apoptotic bodies (Table 1).

The novel minimal information for studies of extracellular vesicles (MISEV) 2018 was proposed to help defining the subtypes of EVs. It suggests that EV subtypes should be classified depending on their size (<100 nm or <200 nm, small-sized EVs or >200 nm, large- and/or medium-sized EVs), density (low, medium, and high), biochemical composition (positive markers), or origin (foot cell EV, hypoxic EV, large tumor body, and cell) [60].

MSC-derived exosomes express specific endosomal markers (cluster of differentiation (CD)9, CD63, CD81, apoptosis-linked gene 2-interacting protein X, and tumor susceptibility gene 101), but not the negative marker (calnexin) [61]. MSC-derived exosomes are involved in the regulation of cell migration, proliferation, differentiation, and extracellular matrix synthesis [62,63]. Some studies have reported that MSC-derived exosomes may contribute to cartilage regeneration by regulating immune responsiveness, reducing cell apoptosis, and increasing proliferation [64,65,66]. Nevertheless, to date, there has been a paucity of literature regarding MSC-derived exosomes in the treatment of OA. Therefore, the purpose of this manuscript was to review the characteristics of MSC-derived exosomes and their use for the treatment of OA.

## 3. Characteristics of Exosomes

**Figure 1:**
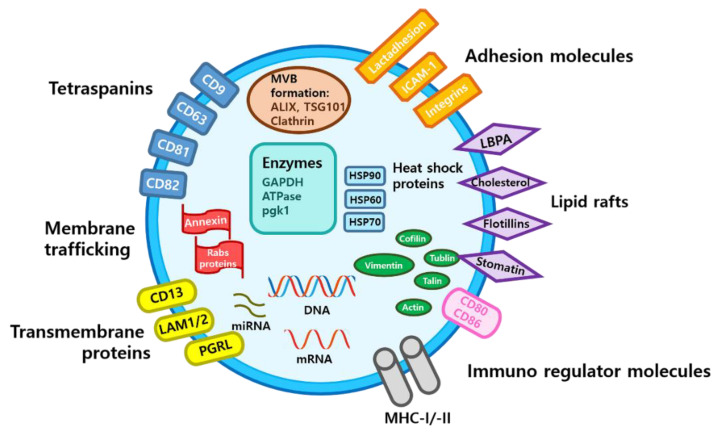
Schematic structure and contents of exosome. ATPase, adenosine triphosphatase; CD, cluster of differentiation; GAPDH, glyceraldehyde 3-phosphate dehydrogenase; HSP, heat shock protein; ICAM-1, intercellular adhesion molecule-1; LAM 1/2, lysosomal-associated membrane protein 1/2; MHC, major histocompatibility complex; miRNA, microRNA; mRNA, messenger RNA; MVB, multivesicular body; PGRL, PG regulatory-like protein; pgk1, phosphoglycerate kinase 1.

Exosomes are nano-sized vesicles (50‒150 nm in diameter) that contain nucleic acids such as micro RNAs and messenger RNAs, functional proteins, and bioactive lipids [67,68,69] (Figure 1).

Exosomes generally mediate cell-to-cell communication and cell signaling, and alter cell metabolism by transferring such functional components [70]. They are released from various types of cells, including B cells, T cells, reticulocytes, dendritic cells, mast cells, epithelial cells, and MSCs [71]. Owing to their transferring ability, exosomes have the potential to stimulate target cells, transfer membrane receptors, deliver proteins, and trigger genetic changes in recipient cells [56,72,73].

Exosomes are released by the endosomal network via intraluminal vesicles (pre-exosomes) formed by inward budding. A clathrin-dependent or clathrin-independent pathway initiates endocytosis at the lipid raft. Exosomal biogenesis occurs through the endosomal network in pathways related to endosomal sorting complexes or independent pathways [74,75]. Endocytic vesicles contain cytoplasmic proteins, a variety of RNA types (messenger RNA, microRNA, transfer RNA, long noncoding RNA, and ribosomal RNA), and nuclear molecules. Different types of RNA in exosomes are involved in the epigenetic modification of cells and alteration of biological activities [76]. Additionally, various bioactive proteins originating from the plasma membrane and cytoplasm also exist in exosomes [77]. The release of exosomes by exocytosis is caused by the fusion of multivesicular bodies and the plasma membrane [78].

Depending on the diameter, exosomes can be classified into two exosome subpopulations (large exosomes, 90–120 nm; small exosomes, 60–80 nm) [79]. Each has different molecular and biological properties. Exomeres are an abundant population with nonmembranous nanoparticles (approximately 30 nm) [79]. They express higher levels of enzymes related to metabolism and hypoxia, and coagulation-related proteins. In contrast, large and small exosomes express more signaling factors related to the mitotic spindle, IL-2/STAT-5, and factors related to endosome secretion pathways [79]. Furthermore, they can be divided into high- and low-density exosomes based on the difference in density gradient centrifugation [80]. Owing to these differences, different molecular and biological effects can be promoted in recipient cells [81].

## 4. Technique for the Isolation of Exosomes from MSCs

The subtypes of EVs that can be categorized by differential sizes have been well documented. The large-size group is relatively heterogeneous and comprises sizes in the range of 90–150 nm, whereas the small-size group is quite homogenous, with sizes of 60–80 nm [82]. According to the size distribution of each subtype of EVs, sedimentation difference, and other specific characteristics, several isolation techniques, including those that are ultracentrifugation-based, size-based, and immuno-affinity action-based, have been introduced and developed. Herein, we discuss different isolation techniques in detail and summarize their strengths and weaknesses [83].

### 4.1. Ultracentrifugation-Based Technique

The isolation of pure exosomes is paramount to investigating their function and role in tissues or disease and conducting further basic or clinical studies. The most popular technique for exosome isolation is the ultracentrifugation method. According to the literature, approximately 80% of researchers have used it for exosome isolation. Generally, considering the characteristics of ultracentrifugation, this technique is a more suitable option for pelleting lipoprotein or extracellular proteins. However, it may not be effective for exosome isolation, owing to its highly labor-intensive and time-consuming nature, low cost-effectiveness, need for large amounts of starting material, and low yields [84]. Meanwhile, density gradient ultracentrifugation was introduced to allow for the further purification of exosomes from dense protein aggregates, contrary to the conventional ultracentrifugation technique [85]. The mechanism of density gradient ultracentrifugation is based on the particle size and density difference between the materials, including cells, EVs (exosomes, microvesicles, and apoptotic bodies), and proteins. After development, subgroups of EVs can differentiate and stay at different layers of the gradient. In other words, refined exosomes, including EVs, can be isolated from whole fluids to enable better basic and clinical studies. Ultracentrifugation technology is considered the gold standard for exosome isolation, with several advantages over other techniques, including low cost and easy application; however, it also has some limitations, such as expensive equipment, laboriousness, long run times, and contamination issues (especially albumin contamination) [86]. In addition, high centrifugal forces can be linked to the demolition of the vesicle structure, which disturbs the study progress and outcomes [87].

### 4.2. Size-Based Technique

EVs consist of microvesicles, apoptotic bodies, and exosomes, and their classification is based on the size of each particle. The exosome diameter (50–150 nm) is smaller than that of other EVs, and this is the fundamental basis of size-based techniques for isolation [84]. To date, several size-based techniques have been widely used in the form of sequential filtration (e.g., ultrafiltration (UF), size-exclusion chromatography (SEC), and size-dependent microfluidics). These methods, especially UF, are rapid, less laborious, relatively inexpensive, and faster than ultracentrifugation. The UF method is a major option with several merits, but it has a significant demerit in that it is difficult to separate other contaminants from exosomes, especially proteins such as albumin [88]. SEC, another size-based technique, involves filtration through a porous stationary phase [89]. There are many pores with a variety of pore sizes, enabling the separation of specific particles, such as exosomes, from proteins. With advances in the technology, the SEC technique has improved gradually, and in combination with other techniques, such as column chromatography, allows the isolation of certain particles, such as exosomes, under fluidic conditions. However, size-based methods using filters have several limitations as well, including a small yield of isolation [42].

Other techniques similar to size-based methods, including the precipitation method, have been developed. Precipitation involves the collection of exosomes by capturing them with “polymer nets” of certain sizes. This technique is simple, rapid, easy to use, and inexpensive for the equipment setup. Moreover, this technique allows easy integration into the clinical field and can produce a larger volume of EVs in a relatively short time, compared with other techniques. However, some issues should be addressed prior to clinical use, including the toxicity of polymers used for the formation of nets and contamination with particles of similar sizes (e.g., protein aggregates and other types of EVs) [86].

### 4.3. Immunoaffinity Interaction-Based Technique

Exosome membranes consist of specific structures and contain large quantities of proteins. The immuno-affinity method is based on the interactions between these proteins, such as antigens, and their antibodies, as well as specific connections between receptors and their ligands [90]. In body fluid, exosomes mingle with other materials, such as various cells, MSCs, and proteins; hence, isolating pure exosomes from the population is difficult, and most isolation methods described above face contamination issues. To address this limitation, immuno-affinity-based techniques have been developed; they can be used to selectively capture specific exosomes from a mixed population [91]. Specific antibodies to certain surface markers of exosomes, such as anti-CD63 and anti-CD9, are widely used for the isolation of pure exosomes. Tauro et al. [88] demonstrated that the immuno-affinity method is more efficient than the ultracentrifugation-based technique for isolating exosomes from colon cancer cells.

## 5. Limitations of MSC-Based Therapies

MSCs have the potential to treat joints with OA; therefore, they have been widely studied, regardless of their origin, over the last few years [11,15,16,92]. However, there are some problems with the approach using MSCs. Although direct cell transplantation is performed, cell survival is difficult to predict after cell injection to sustain the effects due to intercellular interactions, and to maintain adequate cellular storage for commercialization [29]. The longevity issue of MSCs may be associated with improper environmental conditions for MSC survival and poor cell–cell communication between specific cells [32]. In addition, the differentiation capacity of cells may be affected by the condition of the donors. Senescence and loss of proliferative potential can reduce the capacity for differentiation. Kretlow et al. [93] reported that donor age and cell passage affect the differentiation potential of bone marrow-derived MSCs (BM-MSCs). In addition, during the initial phase after MSC implantation, there sometimes exists a paradoxical period in which pro-inflammatory activity dominates over anti-inflammatory activity [94]. In other words, MSCs induce pro-inflammatory activity for attracting innate cells rather than promoting anti-inflammatory activity [35]. Therefore, during this period, MSCs do not survive well, and their function may decline. Moreover, because MSCs are sensitive to the surrounding environment, they can have a negative impact on diseased joint environments such as OA. Lee et al. [95] demonstrated that exposure of human adipose tissue-derived MSCs (AD-MSCs) to TNF-α during inflammation can enhance the inflammatory response. Moreover, in environments of senescence and loss of proliferative capacity, in vitro expansion of MSCs may also be affected before transplantation [96]. Although several studies on OA treatment with MSCs have reported on short-term therapeutic effects, the lack of standardization for isolation and expansion, problems of yields for acquisition, and difficulties in reaching the target tissues of injected MSCs (clearance or catching) remain unsolved [21,22,23].

## 6. MSC-Derived Exosomes and Osteoarthritis

MSC-derived exosomes have been used to overcome the limitations of MSCs. MSC-derived exosomes have advantages including small dimensions, low immunogenicity, and lack of requirement for additional procedures for culture expansion or delivery.

Although the homogeneity in prior studies was poor due to problems with the control group, dose, frequency, and timing, MSC-derived exosomes showed therapeutic effects similar to those of MSCs in preserving tissues and promoting functional recovery from injury [97,98,99]. MSC-derived exosomes increase the expression of chondro-protective markers (collagen type II and aggrecan), reduce the levels of catabolic markers (MMP-13, a disintegrin and metalloproteinase with thrombospondin motif 5 (ADAMTS5)), delay chondrocyte apoptosis, and block macrophage activation [57]. Moreover, they can alleviate the effects of OA by stimulating chondrocyte migration and proliferation [100]. Particularly in OA conditions, MSC exosomes enhance sulfated glycosaminoglycan (s-GAG) matrix synthesis inhibited by IL-1β, and inhibit inducible nitric oxide synthase (iNOS) and MMP13 production induced by IL-1β. In addition, these actions are partially blocked by inhibitors of adenosine receptor activation, protein kinase B (AKT), extracellular signal-regulated kinase (ERK), and adenosine monophosphate-activated protein kinase (AMPK) phosphorylation [101]. Moreover, upregulated microRNAs from MSC-derived exosomes combined with chondrogenic induction may play an important role in exosome-mediated cartilage regeneration [102]. Tofiño-Vian et al. [103] reported that human AD-MSC-derived exosomes exerted chondro-protective functions through multiple mechanisms, including the suppression of inflammatory mediators, decreased MMP activity, and promotion of anti-inflammatory cytokines. They suggest that AD-MSC-derived exosomes can be utilized for the development of new therapeutic approaches for joint conditions.

## 7. Review of Current Studies with MSC-Derived Exosomes for OA Treatment

The functions and properties of MSC-derived exosomes may vary depending on the cellular source of MSCs. Some studies have shown that the contents of exosomes extracted from human AD-MSCs and BM-MSCs, such as RNAs, significantly differ in composition [81,104]. These intrinsic differences between different MSC-derived exosomes can have a significant influence on the therapeutic effects [105,106]. Moreover, exosomes are composed of a variety of biological molecules, such as proteins, nucleic acids, and lipids, which reflect the cellular origin and pathological or physiological conditions of origin cells [107,108]. Hence, although the studies on OA treatment using exosomes reported to date are heterogeneous, most studies reported favorable results in improving the OA environment (Table 2).

Some authors have compared the functions of exosomes and microvesicles in OA. Cosenza et al. [57] reported that there is no significant difference in the functions of BM-MSC-derived exosomes and microvesicles. Interestingly, they found that the highest doses of BM-MSC-derived exosomes or microvesicles could not only reverse the phenotype of chondrocytes, but also regulate anabolic and catabolic chondrocyte markers to a similar degree as that by BM-MSCs. They also showed that pre-activation of BM-MSCs with TGF-β can enhance the efficacy of exosomes and microvesicles. Pre-activated BM-MSC-derived exosomes and microvesicles exhibited significantly different gene expression patterns [16]. Another study showed the anti-fibroblastic function of BM-MSC-derived exosomes pre-activated with TGF-β3 [109]. Reinforcing exosome content through the pre-activation of MSCs with growth factors would be a promising therapeutic option to maximize the anti-osteoarthritic potential of exosomes. One study using AD-MSCs indicated that AD-MSC-derived exosomes downregulate senescence-associated β-galactosidase activity and the accumulation of γH2AX foci. It demonstrated the correction of abnormal osteoblast metabolism by AD-MSC-derived exosomes in a chronic inflammation environment including cellular senescence and joint degradation [110]. Zhang et al. [101] demonstrated the ability of exosomes to repair OA in their study using embryonic stem cell-derived MSCs. It was characterized by the suppression of pain, reduced inflammation, and gradual improvements in matrix expression.

Although several studies have demonstrated the comparable potential of MSC-derived exosomes and MSCs and suggested their potential as cell-free treatment tools for OA, most such studies were conducted only at the laboratory level. Therefore, it is necessary to evaluate the clinical effectiveness of MSC-derived exosomes in patients with OA in clinical settings.

**Table 2:** Details of studies on MSC-derived extracellular vesicles in osteoarthritis.

Authors	Publications /Year	Type of Disease	Type of Cells	Type of EV	Model	Cargo	Function	Additional Manipulation
Tofiño-Vian et al. [103]	Cell Physiol Biochem 2018	OA	AD-MSCs	Exosomes Microvesicles	*In vitro*	Proteins	TNF-α↓ IL-6↓ PGE_2_↓ NO↓ MMP-13↓	Chondrocyte stimulated with IL-1β
Tofiño-Vian et al. [110]	Oxid Med Cell Longev 2017	OA	AD-MSCs	Exosomes Microvesicles	*In vitro*	Proteins	β-galactosidase↓ γH2AX foci↓ IL-6↓ PGE_2_↓	Osteoblast stimulate with IL-1β
Cosenza et al. [57]	Sci Rep 2017	OA	BM-MSCs	Exosomes Microvesicles	Mice	Proteins	Type II collagen↑ Aggrecan↑ MMP-13↓ ADAMTS5↓ iNOS↓	
Sun et al. [102]	J Cell Biochem 2019	OA	BM-MSCs	Exosomes	*In vitro*	microRNA-320c	Chondrocyte proliferation↑ MMP-13↓ SRY-Box 9↑	
Qi et al. [111]	In vitro Cell Dev Biol Anim 2019	OA	BM-MSCs	Exosomes	Rabbit	Proteins	Chondrocyte apoptosis↓ (Phosphorylation of p38↓, ERK↓, AKT↑)	Induction of chondrocytes apoptosis with IL-1β
Mao et al. [112]	Stem Cell Res Ther 2018	OA	BM-MSCs	Exosomes	Mice	microRNA-92a-3p	Cartilage development and homeostasis (direct targeting of WNT5A)	
Zhu et al. [100]	Stem Cell Res Ther 2017	OA	iPSC-MSCs vs. SM-MSCs	Exosomes	Mice	CD9 CD63 TSG101	iPSC-MSCs-exosomes have greater therapeutic effect on OA	Comparative study Intra-articular injection
Zhang et al. [101]	Biomaterials 2019	OA	ESC-MSCs	Exosomes	Rat	Proteins	s-GAG synthesis impeded by IL-1β↑ IL-1 induced NO and MMP-13↓	
Wang et al. [113]	Stem Cell Res Ther 2017	OA	ESC-MSCs	Exosomes	Mice	Proteins	Type II collagen & aggrecan↑ ADAMTS5 with IL-1 β↓	

ADAMTS5, a disintegrin and metalloproteinase with thrombospondin motifs 5; AD-MSCs, adipose tissue-derived mesenchymal stem cells; Akt, protein kinase B; BM-MSCs, bone marrow-derived mesenchymal stem cells; CD, cluster of differentiation; ERK, extracellular-signal-regulated kinase; ESC-MSCs, embryonic stem cell-derived mesenchymal stem cells; γH2AX, phosphorylated H2A histone family member X protein; IL-1β, interleukin-1β; IL-6, interleukin-6; iNOS, inducible nitric oxide synthase; iPSC-MSC, induced pluripotent stem cell-derived mesenchymal stem cells; MMP-13, matrix metalloproteinase-13; NO, nitric oxide; OA, osteoarthritis; PGE_2_, prostaglandin E_2_; s-GAG, sulfated glycosaminoglycan; SOX9, sex-determining region Y-box transcription factor 9; SM-MSCs, synovial membrane-derived mesenchymal stem cells; TNF-α, tumor necrosis factor-α; TSG101, tumor susceptibility gene 101; WNT5A, Wnt Family Member 5A.

## 8. Concluding Remarks and Perspectives

Exosomes perform a wide variety of functions, including the repair of damaged tissues, regulation of immune responses, and reduction in inflammation. Exosomes are multipotent biological mediators extracted from cultured MSCs. MSC-derived exosomes have become very popular in regenerative medicine owing to their ability to repair damaged tissues. MSC-derived exosomes are a type of mediator of cell-to-cell communication via the transfer of RNAs, lipids, and proteins to other cells. Several studies have shown that MSC-derived exosomes have capabilities comparable to those of MSCs. They have demonstrated that MSC-derived exosomes are suitable alternatives to overcome the limitations of existing MSCs.

Although several studies on the use of MSC-derived exosomes as a treatment tool for OA have been performed, there is still a lack of evidence [114]. Efforts to establish a consensus are needed to define the therapeutic potency of MSC-derived exosomes [23]. Further research is needed to prove these outcomes. Moreover, the clinical and radiologic effects of MSC-derived exosomes on OA should be confirmed through clinical studies. Furthermore, it is necessary to investigate the effect of MSC-derived exosomes on chondrogenesis, the ultimate goal of OA therapy, through interaction with other chemo-attractive mediators.

## Figures and Tables

**Table 1:** Summary of details for exosomes, microvesicles, and apoptotic bodies.

	Size (nm)	Morphology	Origin	Pathway Related	Biomarkers	Formation Mechanism	Ultracentrifugation Isolation (× *g*)	Contents
Exosomes	50~150 nm, regular	Cup-shaped	Endosomal compartment of cells, Multivesicular body (MVB)	ESCRT-dependent Tetraspanin-Ceramide-	CD9,63,81 TSG101 Tetraspanins, ALIX, HSP70s	Exocytosis of MVB	100,000~200,000	mRNA, miRNA, IncRNA, protein, lipid, rarely DNA
Microvesicles	100~1000 nm, Irregular	Heterogenous	Plasma membrane	Ca^2+^-dependent Various	Selectins, Integrins CD40 ligand	Budding from membrane	10,000~60,000	mRNA, miRNA, IncRNA, protein, lipid, rarely DNA
Apoptotic bodies	1000~5000 nm, irregular	Heterogenous	Cells Plasma membrace	Apoptosis-related pathway	Histones, Annexin V	Budding from membrane	10,000 (no standardized protocol)	Fragmented DNA, Cell organelles, Nuclear fraction

ALIX, apoptosis-linked gene 2-interacting protein X; CD, cluster of differentiation; ESCRT, endosomal sorting complexes required for transport; HSP70, 70 kilodalton heat shock proteins; MVB, multivesicular bodies; TSG101, tumor susceptibility gene 101.

## Data Availability

Not applicable.
